# Microencapsulation of Black Carrot Pomace Bioactive Compounds: Artificial Neural Network Modeling of Cytotoxicity on L929 Fibroblast Cells

**DOI:** 10.3390/gels12010053

**Published:** 2026-01-05

**Authors:** Rumeyse Önal, Derya Dursun Saydam, Merve Terzi, Mehmet Fatih Seyhan

**Affiliations:** 1 Department of Food Engineering, Gaziantep University, University Boulevard, 27310 Gaziantep, Turkey; rumeyseonal@gmail.com; 2 Department of Nutrition and Dietetics, Istanbul Yeni Yuzyil University, Topkapı Dr. Azmi Ofluoğlu Campus, 34010 İstanbul, Turkey; merve.terzi@yeniyuzyil.edu.tr; 3 Department of Molecular Biology and Genetics, Istanbul Yeni Yuzyil University, Topkapı Dr. Azmi Ofluoğlu Campus, 34010 İstanbul, Turkey; mehmetfatih.seyhan@yeniyuzyil.edu.tr

**Keywords:** black carrot pomace, phenolic compounds, freeze drying, biocompatibility, artificial neural network

## Abstract

Valorization of black carrot pomace (BCP), an industrial by-product rich in bioactive compounds, was performed using sustainable extraction and formulation approaches. Bioactive compounds were extracted, using water as a solvent, via ultrasonic processing. The resulting liquid extract (BCP-E) was then freeze-dried with a gum Arabic gel system to obtain a powder formulation (FD-BCP). The technological, physicochemical, and bioactive characteristics of both formulations are described. Total monomeric anthocyanin and antioxidant activities (DPPH and ABTS) did not differ substantially (*p* > 0.05), but the liquid extract’s total phenolic content was significantly higher (4.95 mg GAE/g db) than the powder formulation’s (4.46 mg GAE/g db). While FD-BCP had three main hydrophilic phenolic compounds, suggesting partial encapsulation, high-resolution LC-MS analysis identified 21 phenolic compounds in BCP-E, dominated by chlorogenic, quinic, and protocatechuic acids. The development of a stable gum Arabic matrix that maintains the phenolics’ structural integrity was confirmed by SEM and FTIR observations. According to cytotoxicity tests conducted on L929 fibroblast cells, both formulations were biocompatible (>70% viability) and even stimulated cell growth at moderate dosages. Dose- and time-dependent viability patterns were successfully described by Principal Component Analysis and Artificial Neural Network models, highlighting the fact that formulation type is the main factor influencing biological response. Overall, ultrasonic extraction and freeze-drying offer efficient and sustainable strategies for producing stable and bioactive-rich components from black carrot pomace that may be used in functional foods and biomedical products.

## 1. Introduction

Fruit and vegetable processing operations annually generate a large quantity of wastes like pulp, core, rind, peels, seeds, and pomace, being one of the most common food waste-producing activities [1]. Improper management of these wastes may lead to serious economic and environmental problems that may be resolved with adequate utilization methods to be a convenient source for functional food ingredients, including bioactive compounds, dietary fiber, flavorings, and coloring agents [2,3,4]. Among these, black carrot (*Daucus carota* L. ssp. *sativus*) stands out due to its rich content in bioactive compounds–mainly chlorogenic acid, quinic acid, quercetin, and catechin–known for their antioxidant, anti-inflammatory properties [5] and impacts of helping immune response and preventing chronic diseases [6]. The pomace generated after industrial processing of black carrot may contain the bioactive compounds that can be employed for product development strategies. Therefore, recovery of black carrot pomace into valuable products can produce economic value, contribute to public health, and achieve sustainability [7,8].

Consumers have recently increased their demand for products that are nutrient-dense, health-protective, and health-improving benefits. Utilizing black carrot pomace to obtain such products not only meets this demand but also offers innovative opportunities for the development of next-generation products and promotes circular economy applications in diverse industries [2]. Efficiently and environmentally friendly methods for extracting the functional components from black carrot pomace have gained attention. Ultrasonic extraction, a green technology, has become widely accepted compared to solvent-based extractions due to its low energy consumption, rapid extraction time, and ability to use non-toxic solvents like water [7,9]. Ultrasonic wave cavitation facilitates cell disruption during the extraction from black carrot pomace, enabling the safe release of soluble and thermo-labile bioactive compounds [8].

The shelf life stability and transportability of bioactive extracts are critical considerations in product formulation, particularly for functional food and nutraceutical applications. Freeze-drying of bioactive extracts with natural carriers such as gum Arabic has been widely reported as an effective strategy for enhancing physicochemical stability and solubility [10]. However, the current literature remains limited in comprehensively addressing the antioxidant, phenolic, and biological activities of such extracts when evaluated simultaneously in both liquid and powder formulations derived from the same extraction system. In particular, studies integrating sustainable water-based ultrasonic extraction, high-resolution phenolic profiling at the molecular level (LC-MS), and gum Arabic-assisted encapsulation of black carrot pomace are scarce. Moreover, the influence of formulation type on cytotoxic or proliferation-related cellular responses has not been systematically investigated, and dose-time-dependent biological effects have rarely been examined using advanced data-driven approaches. In this context, the application of artificial neural networks (ANNs), as supervised machine learning tools capable of modeling complex, nonlinear, and multivariate relationships, remains largely unexplored for predicting cytotoxic responses of phenolic-rich black carrot formulations. Therefore, the present study aims to evaluate the formulation-dependent physicochemical properties and safety-related biological responses of ultrasound-extracted bioactive compounds from black carrot pomace in both liquid and freeze-dried forms. The extracts were characterized in terms of their phenolic composition using high-resolution LC-MS, antioxidant capacity, and physicochemical properties, and their cytotoxic responses in fibroblast-like cell lines were experimentally assessed and further modeled using ANN to provide a comprehensive experimental-computational understanding of formulation-dependent technological and biological interactions.

## 2. Results and Discussion

### 2.1. The Characteristics of the Products

#### 2.1.1. Antioxidant Properties

The only statistically significant difference between the liquid and freeze-dried forms of black carrot pomace was observed for total phenolic content (TPC) (*p* = 0.025). The BCP-E sample exhibited a TPC value of 4.95 mg GAE/g db, while the FD-BCP sample showed a slightly lower value of 4.46 mg GAE/g db. In contrast, no significant differences were detected between the two formulations in terms of antioxidant capacity as measured by DPPH and ABTS assays, nor in total monomeric anthocyanin content (Table 1). This apparent discrepancy between TPC and antioxidant activity can be attributed to the heterogeneous contribution of different phenolic classes to radical scavenging capacity. Total phenolic content reflects the cumulative concentration of phenolic compounds but does not account for differences in molecular structure, reducing power, or antioxidant efficiency. High-resolution LC–MS analysis demonstrated that although phenolic diversity markedly decreased after freeze-drying, key phenolic acids with high antioxidant potential, particularly quinic acid and chlorogenic acid, were largely preserved in the encapsulated powder. These compounds are known to exhibit strong radical scavenging activity and may therefore sustain overall antioxidant capacity despite a reduction in total phenolic concentration. In addition, phenolic compounds with higher molecular weight or lower reducing ability may contribute disproportionately to TPC values while interacting weakly with DPPH and ABTS radicals, as previously reported for structurally complex phenolics with limited electron-donating capacity [11]. Such compounds may also be partially entrapped within the gum Arabic matrix, limiting their analytical accessibility without necessarily compromising functional antioxidant performance. The protective role of gum Arabic against anthocyanin degradation under thermal and oxidative conditions further supports the observed stability of antioxidant capacity, consistent with its well-established encapsulating properties [12].

#### 2.1.2. Technological Properties

In addition, the liquid form of black carrot pomace extract exhibited a pH of 3.50 ± 0.014 and °Brix of 1.04 ± 0.028, indicating a highly acidic and dilute aqueous system characteristic of anthocyanin-rich matrices. The color results of both samples were statistically significantly different from each other (Table 1). The color evaluation revealed distinct variations between the liquid and freeze-dried forms. The liquid extract showed very low lightness and high yellowness (L* = 3.76 ± 0.20; a* = 9.76 ± 0.39; b* = 2.74 ± 0.49; YI = 136.43 ± 1.16), indicating a dark purplish-red tone typical of concentrated anthocyanin systems. In contrast, the freeze-dried powder showed much higher lightness and lower YI values (L* = 60.92 ± 0.02; a* = 14.07 ± 0.02; b* = −4.10 ± 0.01; YI = 7.89 ± 0.03), reflecting color brightening and pigment protection within the gum Arabic matrix formed during freeze-drying.

The physicochemical profile of the encapsulated powder indicates a generally stable and process-friendly formulation suitable for further food and pharmaceutical applications. The measured glass transition temperature (Tg) of 52.59 ± 0.42 °C reflects adequate thermal stability, suggesting that storage and processing conditions should remain below this threshold to preserve structural integrity and prevent undesirable physical transitions [13]. From a flow properties, the low bulk density (0.11 ± 0.002 g/cm^3^) and tapped density (0.12 ± 0.002 g/cm^3^), together with a Hausner ratio of (1.11) Carr index (10%), indicate acceptable flow characteristics that are advantageous for transport, blending, and uniform dosing in industrial applications [14], although the expanded particle structure associated with low density may increase packaging volume requirements and enhance susceptibility to oxidative degradation due to greater interparticle air spaces. According to flowability indices, Hausner ratio (HR ≤ 1.11) and Carr index (CI ≤ 10%) values indicate excellent flow characteristics, which are advantageous for large-scale processing, packaging, and transportation [15]. Moisture-related properties further support formulation stability, as the moderate hygroscopicity (15.6 ± 0.44%) combined with a low moisture content (3.66 ± 0.42%) suggests controlled moisture uptake, contributing to both product stability and reconstitution performance, while also highlighting the need for moisture-barrier packaging to minimize agglomeration during storage [16]. Consistently, the measured water activity (aw = 0.169 ± 0.005) remained well below the critical threshold for microbial growth, confirming the formulation’s microbiological and storage stability. The Aw values in the powder stayed below 0.2, which is deemed appropriate for suppressing undesirable microbial development and minimizing biochemical breakdown [16]. The high solubility (88.54 ± 1.99%) reflects efficient water interaction of the gum Arabic matrix, facilitating dispersion in aqueous systems and incorporation into beverages, dairy matrices, and reconstituted dry mixes. In contrast, the relatively long wettability time (296 ± 5.66 s) indicates delayed initial water penetration, which may limit suitability for instant beverage formulations or other applications requiring rapid rehydration. This behavior can be attributed to the compact and dense surface morphology observed by SEM, which reduces the availability of open channels for water entry at the particle interface. These findings are consistent with previous reports on plant extract-based encapsulated powders, which emphasize that low moisture content, high solubility, and favorable flow properties are key to maintaining functional integrity and extending shelf life. The encapsulated formulation, therefore, exhibits desirable technological characteristics supporting its potential application in both food and pharmaceutical delivery systems.

#### 2.1.3. Image Under SEM

**Figure 1:**
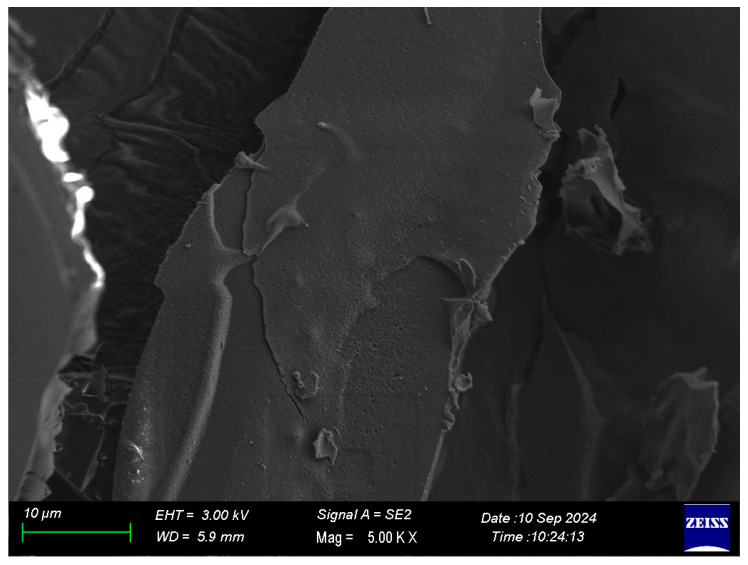
SEM image of the freeze-dried sample.

The SEM micrograph of the freeze-dried black carrot pomace extract with gum Arabic (Figure 1) exhibited a compact and continuous surface, with the presence of smooth plate-like particles; minimal porosity and no visible cracks indicated the formation of a stable and cohesive matrix during freeze-drying. This structural density/compactness supports that components of the extract were integrated into the gum Arabic gel system rather than just deposited on the surface of the particles, which is a typical structure of freeze-dried systems in which slow sublimation allows the formation of dense matrices with low structural collapse [17]. The compact and dense structure of the particles could also be preferred for the encapsulated phenolic compounds’ improved stability through reducing oxygen and moisture diffusion. Contrarily, the denser compact matrix could also limit rehydration efficiency due to excess moisture entering through the particles’ surfaces. The findings confirm that freeze-drying allowed the initiation of a continuous encapsulation matrix that was able to protect and maintain the extract during storage.

#### 2.1.4. Image Under FTIR

**Figure 2:**
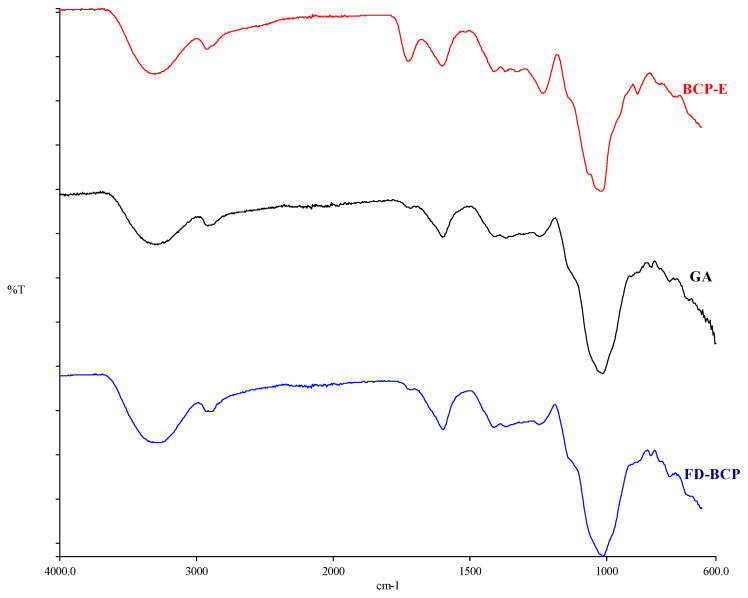
FTIR result of black carrot extract, gum Arabic, and freeze-dried black carrot pomace.

The FTIR spectra supported the morphological observations (Figure 2). Both BCP-E and freeze-dried encapsulated powder showed characteristic absorption bands of gum Arabic and phenolic compounds, indicating that the main chemical structures of both remained intact after drying. The broad peak from 3200 to 3400 cm^−1^ was attributed to O-H stretching vibrations of hydroxyl groups, while the band close to 2920 cm^−1^ belonged to C-H stretching of aliphatic groups [18]. Signals that were detected between 1600 and 1700 cm^−1^ corresponded to aromatic C = C and carbonyl (C = O) stretching of phenolic compounds [19]. The similarity in the spectra of BCP-E and FD-BCP indicates that the freeze-drying process does not appear to cause major changes in the chemical structure. Some minor spectral changes within the regions of 1600–1700 and 1000–1200 cm^−1^ could be attributed to hydrogen bonding or weak molecular interactions between gum Arabic and phenolic fraction. These spectroscopic changes reflect physical entrapment rather than a chemical interaction between core and wall materials. Generally, the SEM and FTIR findings confirmed that the freeze-drying process preserved the structural and chemical integrity of the gum Arabic and black carrot pomace extract system. The compact shape revealed by SEM demonstrates a fortified encapsulation structure. The FTIR outcomes show that the composite legal groups did not change, implying that the encapsulation was successful and bioactive compounds present in the extract remained intact.

### 2.2. Identification of Bioactive Substances

Table 2 presents the results of LC-MS analysis. In the extract of black carrot pomace (BCP-E), 21 different phenolic compounds were determined. This result indicates that this extract has a high flavonoid and phenolic acids-rich compositional profile. Ultrasonication is thought to provide favorable outcomes in this respect, as Nirmal et al. [20] have also presented. The highest concentration of main constituents includes liquiritigenin (1567.696 ng/mL), quinic acid (1692.212 ng/mL), protocatechuic acid (856.257 ng/mL), caffeic acid (384.855 ng/mL), and ferulic acid (166.298 ng/mL). This suggests that these constituents are the primary phenolics that provide black carrot pomace its strong antioxidant potential. Hydroxybenzoic and hydroxycinnamic acid derivatives are particularly important for scavenging free radicals and mitigating oxidative stress. Additionally, phenolic acids contribute to the improvement of biological activity in conjunction with flavonoid derivatives (such as luteolin, naringenin, and liquiritigenin) [21,22]. Smeriglio et al. [23] applied LC-DAD-FLD-MS/MS analysis to quantify 25 polyphenols in black carrot crude extract, which consisted of anthocyanins (78.06%), phenolic acids (17.89%), and other flavonoids (4.06%). Koley et al. [24] identified a total of 11 compounds from Indian black carrot, which included two flavanols and nine anthocyanins using high-resolution LC-MS. Eleven anthocyanin compounds (colored phenolics) and nineteen colorless phenolics from black carrot pomace were identified using LC-DAD-ESI-MS/MS analysis by Polat et al. [25].

In the freeze-dried sample, only three phenolic compounds were identified: quinic acid (61.773 µg/g), chlorogenic acid (89.649 µg/g), and 2,4-dihydroxybenzoic acid (6.035 µg/g). Comparing this to the BCP-E sample, it can be seen that compound diversity significantly decreased. This may indicate that some of the phenolic compounds were kept inside the matrix of gum Arabic and could not be released during analysis. It is well documented that flavonoid compounds can form complexes via hydrophobic or hydrogen bonding interactions with the high molecular weight polysaccharide structure of gum Arabic, which makes recovery analytically difficult [26,27]. From the high preservation of quinic acid and chlorogenic acid, it may be concluded that gum Arabic microencapsulation could have a protective effect on hydrophilic phenolics.

### 2.3. Results of Cytotoxicity Analysis

**Figure 3:**
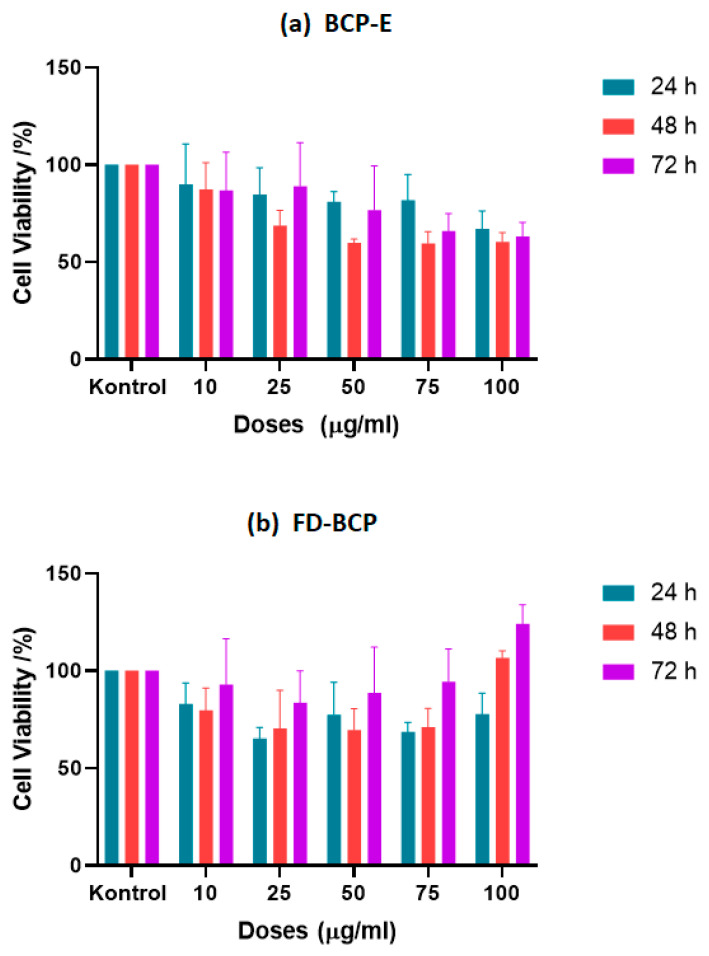
The cell proliferation effect of (**a**) liquid BCP; (**b**) freeze-dried BCP on L929 cell line.

The BCP samples were applied onto L929 cells at a series of concentrations, and cell viability was evaluated using the MTT assay at 24, 48, and 72 h. Measurement of formazan formation indicated that neither of the BCP samples exhibited notable cytotoxic effects across the tested doses and time intervals. The 24 h time point specifically demonstrates the presence of cell viability percentages consistently greater than 75% for all concentrations studied, signifying good biocompatibility at early time points. The IC_50_ value we calculated at this time point was 480 µg/mL and suggests a low cytotoxicity profile overall. At 48 h, we observed a significant change in cell viability depending on the concentration (*p* < 0.05). The IC_50_ value decreased to 153 µg/mL, resulting in moderate sensitivity of cells to higher doses of the liquid extract. Importantly, starting at 50 µg/mL, metabolic activity was significant compared to the control treatment (*p* < 0.05), indicating that while we did observe a concentration-dependent reduction in viability, the extract had more of a mild stimulatory response rather than toxicity. The IC_50_ at the 72 h time point slightly increased to 188.2 µg/mL, and at this time point, we did not observe a further decline in cell metabolic activity, indicating that cells were not negatively impacted due to prolonged exposure to BCP-E. Overall, the BCP-E exhibited a relatively stable biocompatibility profile and suggested a potential proliferative effect at intermediate concentrations (Table 3). Parallel to Table 3 results, Figure 3a demonstrates that the MTT assay results showed that black carrot pomace (BCP) samples were not cytotoxic to L929 cells at any tested concentration or time point (24, 48, and 72 h). Cell viability remained above 75% after 24 h of exposure to BCP-E, indicating good biocompatibility. A minor decrease in viability (to just above 60%) was observed at 48 h with 50 µg/mL BCP-E, but this was not considered toxic. After 72 h, cell viability was similar to that at 24 h, suggesting that extended exposure did not harm the cells. Similar to these results, one study investigated the effects of a combined product based on dense carrot root extract (not black carrot pomace) and quercetin on the L929 cell line. The obtained results showed that the migration and proliferative activity of L929 cells treated with the extract did not have a significant difference from intact cells, which assumes the non-usage of cytotoxic effects of the obtained extract [28].

In the case of the FD-BCP sample, both the 24 and 48 h treatment time points yielded similar results, and cell viability sustained above 60% at all concentrations, suggesting low cytotoxicity. Based on the statistical analyses, cell viability was shown to be significantly better at concentrations between 25 and 75 µg/mL in the first 48 h when compared to the control (*p* < 0.05). Moreover, the freeze-dried formulation did not show any obvious IC_50_ value at any of the time points established, indicating that the freeze-dried formulation is stable and possesses low cytotoxicity. Additionally, a slight decrease in viability was measured at 100 µg/mL at 72 h (*p* < 0.05), which may be more related to cellular adaptation or saturation due to the higher concentration rather than cytotoxicity. Furthermore, exposure to FD-BCP resulted in a measured dose-dependent increase in cell viability at lower concentrations at 72 h, indicating prolonged exposure led to increased metabolism of the fibroblasts at lower concentrations (Table 3). To visualize these results of cell viability and dose-dependent response, Figure 3b shows that the FD-BCP group was above 60% viability at all concentrations at 24 to 48 h, indicating negligible cytotoxicity. Furthermore, at 72 h, a dose-dependent increase in viability was noted, and this was particularly the case for higher concentrations, where cellular metabolic activity was enhanced. The findings indicate that FD-BCP is a harmless treatment and may promote cellular activity following extended exposure. The increased viability may be linked with the high residual phenolic and anthocyanin content from freeze-drying [29]. The particulate nature of the freeze-dried formulation may have also provided a surface for nutrient exchange and cellular adhesion and promoted fibroblast proliferation.

The high-resolution LC-MS analysis provided results indicating that chlorogenic acid and quinic acid were some of the main focused phenolic components in each formulation type, albeit they were also at significantly higher concentrations in the freeze-dried powder formulation. Chlorogenic acid and quinic acid have been evaluated and shown to have effects on cell proliferation using the L929 mouse fibroblast cell line. Chlorogenic acid has been associated with increased fibroblast cell proliferation, with various studies reporting a relatively wide range of increases in fibroblasts, ranging from 23% to 789% [30]. These phenolic compounds are well known for their bioactivity related to influence on fibroblast systems with respect to proliferation, fibroblast proliferation, and extracellular matrix synthesis. Chlorogenic acid has been reported to increase not only fibroblast proliferation, but fibroblasts’ deposition of collagen, thereby promoting wound healing facilitated by the management of oxidative stress and up-regulating genes associated with tissue repair [30,31,32,33]. Similarly, quinic acid has been reported to promote dermal fibroblast activity during tissue regeneration, as well as contribute to cellular migration during tissue regeneration [34]. Quinic acid has also been shown to increase cell growth inhibition in monolayer, particularly when predisposed to treatment, through its modulation of significant pathways, such as MAPK and PI3K/Akt/mTOR [35].

The variations between the two formulations may be attributed to encapsulation offering physicochemical stability. Freeze-drying has been shown to protect anthocyanins and other polyphenolic compounds from oxidative degradation, allowing the bioactivity to be preserved and resulting in a more regulated release of bioactive molecules. In turn, this may enhance cell proliferation through modulation of oxidative balance and stimulation of antioxidant signaling pathways. Other anthocyanin-rich or polyphenol-encapsulated extracts have been reported to support similar trends with slight proliferative effects on fibroblast models [36]. Venezia et al. [36] functionalized silica nanoparticles with pomegranate peel extract and found that encapsulation promoted the stability of phenolic compounds while improving biocompatibility in L929 cells. Moreover, Horani et al. [37] described that polyvinyl alcohol nanofiber mats containing artichoke bracts extract at different concentrations (10, 20 and 40 wt%) produced by electrospinning showed no cytotoxicity to L929 fibroblast cells, supporting biocompatibility. Cell viability was still above 90% at moderate concentrations (≤50 µg/mL), with only mild signs of cytotoxicity observed at the higher concentrations (100 µg/mL). On the basis of this evidence, a regulated release of encapsulated polyphenols may maintain a redox balance while supporting cell viability. These findings are consistent with the current study and show that the freeze-dried BCP formulation was more stable and retained bioactivity compared to the liquid extract.

Both BCP-E and FD-BCP exhibited good biocompatibility to L929 fibroblasts. While BCP-E maintained a consistent viability profile throughout the time points, FD-BCP displayed specificity at defining longer exposures, especially at higher doses, of stimulating cell viability. These data lend support to the utility of both formulations as safe biomaterials with the FD-BCP possibly providing a further advantage of positively enhancing cellular responses over time. According to ISO 10993-5 [38], both formulations were non-cytotoxic, with a >70% viability level, indicating both materials were low in cytotoxicity and demonstrated good biocompatibility in the tested range [39].

#### Modeling Results of PCA and ANN

Evaluation of PCA results

**Figure 4:**
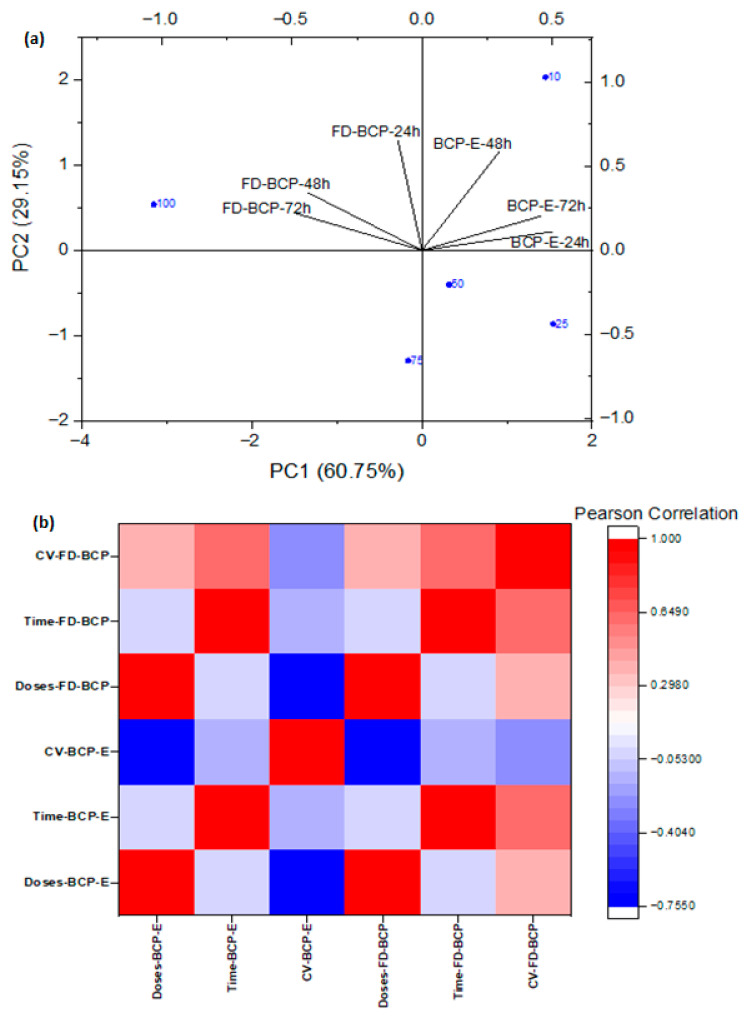
Cell viability responses in a clear dose- and time-dependent manner for BCP-E and FD-BCP: (**a**) PCA results; (**b**) heatmap depiction.

The Principal Component Analysis (PCA) showed that cell viability responses were influenced in a clear dose and time-dependent fashion following treatment with BCP-E and FD-BCP (Figure 4a). As the dose markers distributed along the first principal component (PC1), the dose and response relationship was demonstrated, whereby the lower concentrations, specifically 10 µg/mL, were clustered together near areas of higher viability, while the intermediate and higher doses (50–100 µg/mL) were found clustered at locations along PC1 that were least associated with higher viabilities, which was similarly observed in the MTT assay at 48 h with BCP-E. The secondary principal component (PC2) reflected temporal changes, with some of the further separation occurring at 72 h, with the FD-BCP sample having a dose-dependent increase in viability at the higher concentrations compared to lower concentrations, whereas BCP-E exhibited a response pattern more consistent with 24 h. Additionally, BCP-E and FD-BCP samples were located in distinct regions of PCA space, drawing emphasis to the fundamental role of the type of formulation (liquid vs. powder) on cellular outcomes. The observed distinction is consistent with the MTT assay results. The liquid formulation maintained viability across all tested doses with only minor reductions under specific conditions, whereas the powder formulation exhibited a delayed and dose-dependent stimulatory effect following prolonged exposure (72 h). This separation could be due to release and stability differences in the bioactive compounds in liquid and freeze-dried forms. In general terms, the PCA was a strong indicator that the bioactive nature of the black carrot pomace is indicative of concentration and time sequencing, but in addition, included type of formulation. BCP-E and FD-BCP had opposing effects on cell viability.

The Pearson correlation analysis highlighted distinct dose- and time-dependent responses of BCP-E and FD-BCP. In BCP-E, cell viability (BCP-E) showed a negative correlation with dose (Doses-BCP-E), consistent with the modest reductions observed at intermediate concentrations, while correlations with time (Time-BCP-E) were weak. In contrast, FD-BCP displayed strong positive correlations of viability (FD-BCP) with both dose and time, indicating enhanced cell survival at higher concentrations and prolonged exposure, in agreement with MTT results at 72 h. Overall, the heatmap supports PCA findings by demonstrating that formulation type drives divergent outcomes: the liquid extract maintained stable viability, whereas the freeze-dried powder promoted a dose- and time-dependent increase in cell survival (Figure 4b).

Evaluation of ANN results

**Figure 5:**
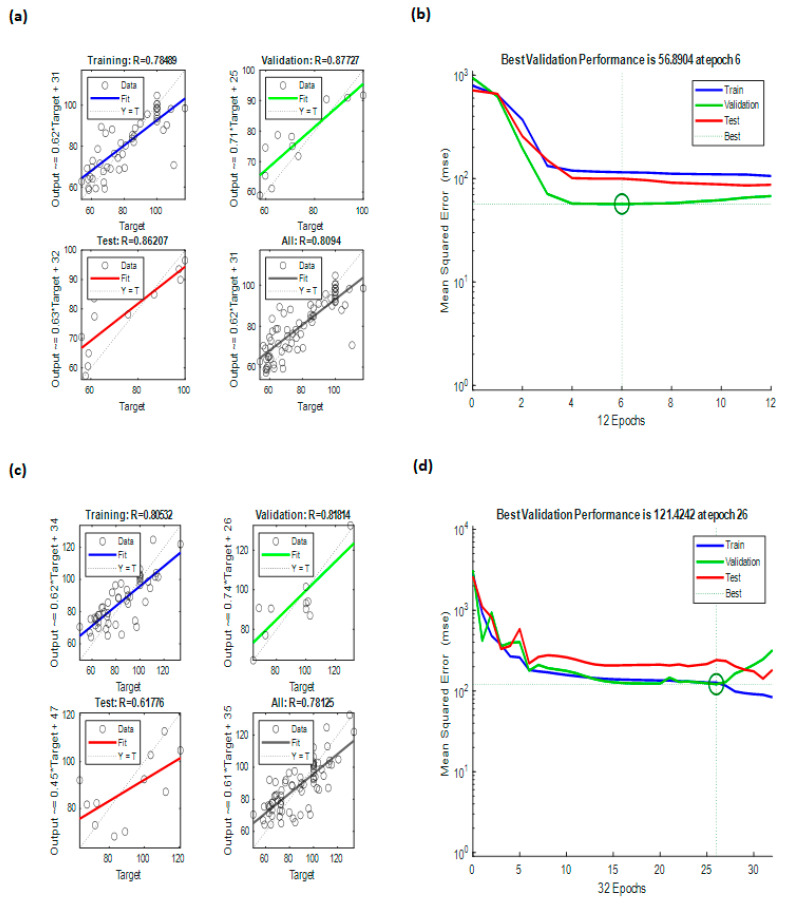
ANN modeling results: (**a**) regressions of ANN modeling for BCP-E, (**b**) best validation performance of ANN for BCP-E, (**c**) regressions of ANN modeling for FD-BCE, and (**d**) best validation performance of ANN for FD-BCP.

The ANN models demonstrated reliable predictive ability for cell viability responses as a function of dose and incubation period/exposure time. For the liquid extract, the model achieved a correlation coefficient of R = 0.8094 (Figure 5a), while the powder extract yielded R = 0.78125 (Figure 5c), indicating good agreement between predicted and experimental values in both cases. As previously mentioned, the liquid extract was modeled with slightly better accuracy which suggests that its biological responses were more consistent to be represented by the nonlinear structure of the ANN. This was confirmed through the mean squared error (MSE) plots that consistently produced lower prediction errors for the liquid model (Figure 5b) compared to the prediction errors reflected for the powder model (Figure 5d). This suggests that there was a reasonable lack of prediction reliability in the powder extract model when compared to the liquid extract model, which was also found in the regression plots.

The stronger predictive capabilities of the liquid formulation may be associated with the differences in the distribution and availability of bioactive compounds compared to the powder, which likely produced more variability in the cellular responses. The ANN analysis demonstrated that models of both formulations followed predictable patterns of dose–response and time dependency, and the liquid extract model exhibited relatively higher robustness than the powder extract model overall. These results support the ANN as a useful analytical tool to model complex bioactivity data in cell viability systems.

## 3. Conclusions

This study presents a comprehensive experimental–computational framework for the valorization of black carrot processing waste through sustainable water-based ultrasonic extraction, formulation-dependent stabilization, and biological safety assessment. Unlike previous studies that primarily focused on extraction efficiency or antioxidant activity alone, the present work provides an integrated comparison of liquid extract (BCP-E) and freeze-dried encapsulated powder (FD-BCP) obtained from the same extraction system, thereby revealing formulation-driven differences in physicochemical behavior and biological response. From an application standpoint, the liquid extract (BCP-E) exhibited a diverse phenolic profile and high antioxidant activity, making it suitable for applications requiring rapid dispersion or direct incorporation into aqueous food matrices. In contrast, the freeze-dried encapsulated formulation (FD-BCP) offered enhanced physicochemical stability, portability, and storage safety, supported by FTIR and SEM analyses confirming the protective role of the gum Arabic matrix on anthocyanins. These results indicate that the two formulations are complementary rather than interchangeable, with distinct applicability depending on the intended food or nutraceutical application. A key innovation of this work lies in the integration of artificial neural network (ANN) modeling with experimental cytotoxicity data. The ANN approach, as a supervised machine learning tool, successfully captured nonlinear and time-dependent relationships between formulation type, concentration, and cellular response, extending beyond conventional statistical interpretation. This predictive capability provides a practical in silico tool for safety-oriented formulation optimization and supports data-driven decision-making in functional ingredient development. Although the biological evaluation was limited to a single fibroblast cell line and fixed processing conditions, the proposed framework establishes a foundation for future research. Further studies incorporating a wider range of extraction and encapsulation parameters, long-term stability assessment, additional cell models, or in vivo approaches would enhance the translational relevance of the findings. Overall, this study contributes a novel and scalable strategy for developing safe, stable, and application-specific bioactive ingredients from black carrot pomace.

## 4. Materials and Methods

### 4.1. Materials

Black carrot pomace (BCP) was supplied from a black carrot concentrate manufacturer (Erkon Konsantre Co.) located in Konya, Türkiye, and kept at −70 °C until processing and analysis. Gum Arabic was purchased from Kimbiotek Kimyevi Mad. San. ve Tic. Co. (İstanbul, Türkiye). The chemicals of analytical grade from Sigma-Aldrich, the L929 cell line (NCTC clone 929-American Type Culture Collection, Rockville, MD, USA), Dulbecco’s Modified Eagle Medium (DMEM) (EuroClone DMEM Mix F12, Milan, Italy), 10% fetal bovine serum (FBS) (Capricorn Scientific, Ebsdorfergrund, HE, Germany), 1% glutamine (Capricorn Scientific, Ebsdorfergrund, HE, Germany), and 1% penicillin (Capricorn Scientific, Ebsdorfergrund, HE, Germany) were utilized in the experiments.

### 4.2. Extraction Process by Ultrasonication

The BCP was thawed overnight at room temperature before ultrasonic extraction as reported by Santos et al. [40] and Agcam et al. [41] with certain changes. After mixing the BCP and distilled water in a 1:10 (g/mL) ratio, the mixture was ultrasonically treated at 47 kHz and 60 W for 45 min at 60 °C (Branson 2200 Ultrasonic Cleaner, Brookfield, CT, USA). Santos et al. [40] reported that phenolic extraction from pomace is most effective at moderate temperatures (60–70 °C) with extraction times of around 1 h. The extract solution was vacuum-filtered using a Kitasato flask and a Buchner funnel fitted with a glass microfiber filter. After 48 h of freeze-drying (Alpha 1–4 LDplus model, Martin Christ, Osterode am Harz, NI, Germany) at −47 °C, the BCP extract (BCP-E) was stored at 4 °C for further examination.

### 4.3. Microencapsulation Process

Based on preliminary experiments with the working intervals of water and gum Arabic gel, the final microencapsulation solution, which consisting 25% gum Arabic (*w*/*v*), was selected to be employed in the process because of its ability to provide high dry material.

The gum Arabic was dissolved in distilled water and stirred at 500 rpm for an hour using a magnetic stirrer to prepare the microencapsulation gel structure. After 15 min of autoclaving at 121 °C, the gel was cooled to 25 °C and refrigerated for the night. Equal amounts (100 mL) of the BCP extract and the gel were agitated for 30 min at 500 rpm on the magnetic stirrer [19]. As a dryer feed solution, the BCP extract and the gel mixture were first pre-frozen for 24 h at −18 °C, and then freeze-dried under vacuum for an additional 24 h at about −47 °C. Finally, the product in powder form (FD-BCP) was obtained after being dried with a freeze dryer.

### 4.4. Chemical and Analytical Analyses

#### 4.4.1. Moisture Content, pH, and Brix Analyses

Moisture content of FD-BCP sample was determined using an oven (RT 500 W.C. Heraeus Hanau, Hanau, HE, Germany) at 105 °C for 4.5 h [42]. Brix and pH for BCE sample were determined using a digital refractometer (PTR 46X, Index Instruments, Ramsey, Cambridgeshire, UK) and a pH meter (Hanna pH 211, Hanna Instruments, Villafranca Padovana, Italy), respectively.

#### 4.4.2. Color Analysis

Color measurements in terms of lightness/darkness (L*), redness/greenness (a*), and yellowness/blueness (b*) of the samples were measured using HunterLab Colorflex (A60-1010-615 Model Colorimeter, HunterLab, Reston, VA, USA) according to CIELAB system.

#### 4.4.3. Wettability, Hygroscopicity, and Solubility Analyses

The methods described by Jinapong et al. [14], by Cai and Corke [43], and by Cano-Chauca et al. [44] were employed for wettability (%), hygroscopicity (%), and solubility (%) properties of the FD-BCP sample, respectively. Wettability was assessed by measuring the time required for complete wetting of the powder upon contact with water, hygroscopicity was evaluated based on moisture uptake under controlled relative humidity conditions, and solubility was determined from the proportion of soluble solids recovered after dispersion in water.

#### 4.4.4. Density Measurements

With a few adjustments, the density characteristics of the FD-BCP sample were ascertained using the methodology of Asokapandian et al. [45]. The mass of the powder was divided by the volume occupied in the cylinder to determine the bulk density (*pB*) (Equation (1)). After the cylinder was manually tapped 50 times from a height of 10 cm on a bench, the weight of the powder inside was used to determine the tapped density (*pT*) (Equation (2)).

(1) $$p_{B}(g/mL)=\frac{m_{0}}{V_{0}}\times 100$$

(2) $$p_{T}(g/mL)=\frac{m_{0}}{V_{n}}\times 100$$

The flowability and cohesiveness properties of the FD-BCP sample were evaluated in terms of Carr index (*CI*) and Hausner ratio (*HR*), respectively. The following Equations (3) and (4) were used to derive the bulk and tapped density values.

(3) $$CI=\frac{p_{T}-p_{B}}{p_{T}}$$

(4) $$HR=\frac{p_{T}}{p_{B}}$$

#### 4.4.5. Differential Scanning Calorimetry (DSC) Analysis

Using a DSC equipment (DSC-6, Perkin Elmer, Waltham, MA, USA), the glass transition temperature (Tg) of the FD-BCP sample was determined using the methodology described by Beldarrain-Iznaga et al. [46]. Five mg powder was placed in an aluminum pan and heated at a rate of 10 °C/min, between 25 and 200 °C, with a nitrogen flow of 20 mL/min.

### 4.5. Characteristics of Bioactive Compounds

The method of Laureanti et al. [47] was used to extract the phenolics from the samples. A 15 mg portion of the powder was mixed with 3 mL of a solvent containing ethanol, acetic acid, and water (50:8:42, *v*/*v*) for total phenolic compounds, DPPH, and ABTS analyses. For total monomeric anthocyanin content (TMAC), 1 g of powder was mixed with 10 mL of the same solvent. The mixtures were vortexed for 1 min, sonicated for 20 min using an ultrasonic cleaner (Branson 2200, Shelton, CT, USA), and filtered using qualitative filter paper (0.45 μm).

#### 4.5.1. Total Phenolic Compound Analysis

After 5 min mixing of 2.5 mL of Folin-Ciocalteau reagent (0.2 N) and 100 µL diluted sample, 2 mL of sodium carbonate solution (75 g/L) was added to the mixture. Absorbance was measured at 760 nm using a double-beam spectrophotometer (Pharmacia Biotech Novaspec II, Cambridge, UK) following an hour of dark conditions at 25 °C. Gallic acid equivalent (GAE) per gram of sample was used to express the results [27].

#### 4.5.2. Determination of Antioxidant Activity by DPPH Assay

The antioxidant capacity of the samples was determined in terms of Trolox equivalents using the 2,2-Diphenyl-1-picrylhydrazyl (DPPH) method. A 100 µL aliquot of each sample was mixed with 3.9 mL of DPPH solution (0.6 mM) and incubated in the dark for 1 h. Absorbance was measured at 517 nm, and antioxidant activity was expressed as Trolox equivalents (mg TE/g sample) based on a calibration curve generated with Trolox [48].

#### 4.5.3. Determination of Antioxidant Activity by ABTS Assay

The radical scavenging capacity of the samples was determined in terms of Trolox equivalents using the ABTS method [49]. A 40 µL aliquot of each sample was mixed with 4 mL of ABTS radical solution and incubated in the dark for 6 min. Absorbance was measured at 734 nm, and antioxidant activity was expressed as Trolox equivalents (mg TE/g sample) using a Trolox calibration curve.

#### 4.5.4. Determination of Total Monomeric Anthocyanin Content

The total monomeric anthocyanin content (TMAC) was estimated using the pH differential method [50]. The sample was diluted with pH = 1.0 (0.025 mol/L KCl) and pH = 4.5 (0.4 mol/L CH_3_CO_2_Na) buffers to achieve the same dilution. The absorbance was measured at 515 nm and 700 nm in both pH = 1.0 and pH = 4.5 buffers and calculated as in Equation (5).

(5) $$A={\left(A_{515}-A_{700}\right)}_{pH=1.0}-{\left(A_{515}-A_{700}\right)}_{pH=4.5}$$

where *A*_515_ is the absorbance at 515 nm, and *A*_700_ is the absorbance at 700 nm. Then, TMAC (expressed in terms of cyanidin-3-glucoside) was calculated using the following formula (Equation (6)).

(6) $$TMAC\;\left(mg/L\right)=(A\times MW\times DF\times 1000)/(\varepsilon \times 1)$$

where *A* is the absorbance calculated in Equation (5), *MW* is the molecular weight of cyanidin-3-glucoside (449 g/mol), *DF* is the dilution factor, *ε* is the molar extinction coefficient of cyanidin-3-glucoside (26,900). The TMAC in the sample was quantified in terms of cyanidin-3-glucoside equivalents in the unit of mg per g sample.

#### 4.5.5. Liquid Chromatography High-Resolution Mass Spectrometer Analysis

Sample preparation:

10 mg of the FD-BCP sample was weighed and dissolved by adding 5 mL of methanol and 5 mL of a solution containing 0.5% acetic acid. The BCP-E sample (without dilution) was filtered using a 10 mL syringe through a 25 mm diameter PTFE syringe filter with a pore size of 0.22 μm and placed in a 15 mL falcon tube. 1.5 mL of this solution was placed in a vial, shaken thoroughly with a vortex, and then injected into the Liquid Chromatography High-Resolution Mass Spectrometer (LC-HRMS) device [51].

For the determination of compounds to be determined by LC-Orbitrap HRMS, different concentrations of standard phenolic substances (10 ppb, 20 ppb, 40 ppb, 60 ppb, 80 ppb, 100 ppb, 200 ppb, 400 ppb, 600 ppb, 800 ppb, and 1000 ppb) were prepared and each injection was made in three replicates.

Chromatography and High-Resolution MS conditions:

LC-HRMS analyses were performed using an Exactive Plus Orbitrap (Thermo Fisher Scientific, Waltham, MA, USA) high-resolution MS system with a heated electrospray ionization interface and equipped with a pump, an autosampler, and a column oven (DIONEX UltiMate 3000 RS, Sunnyvale, CA, USA). The Orbitrap-MS instrument was calibrated with positive and negative calibration solutions (Pierce™ LTQ Velos ESI Positive Ion Calibration Solution, Waltham, MA, USA) using an automatic syringe injector (Thermo Fisher Scientific, Waltham, MA, USA).

In the LC-HRMS analyses, the LC and MS parts were run simultaneously with the TraceFinder 3.2 program (Thermo Fisher Scientific, Waltham, MA, USA) installed on the system computer, and the data were collected and recorded with the Xcalibur software Version 2.1.0.1140 program (Thermo Fisher Scientific, Waltham, MA, USA).

A 3C18-EB 100 mm × 2 mm UHPLC (COSMOSIL, Nacalai Tesque Inc., Kyoto, Japan) column was used in the analyses. The column oven temperature was set at 30 °C. The elution gradient consisted of 0.5% (*v*/*v*) glacial acetic acid prepared in ultrapure water obtained with the Ultrapure water system (GFL 2004/Human power 1, Burgwedel, Lower Saxony, Germany) in the mobile phase A, and 99.9% pure LC-MS grade methanol in the mobile phase B. Chromatographic separation was carried out under gradient elution conditions with a sample injection volume of 20.0 μL and a flow rate of 0.3 mL/min. The total analysis time was set as 20 min.

The Orbitrap HRMS was operated in both positive and negative modes (Full MS/AIF modes). The ionization interface was set to a sheath gas flow rate of 35; an auxiliary gas flow rate of 7; a spray voltage of 3.5 kV; a capillary temperature of 350 °C; an auxiliary gas temperature of 350 °C; and an S-lens RF level of 50. The MS scan range was 60–800 *m*/*z*; a resolution of 17,500; an ACG target of 3.106; a maximum IT of 2 ms; and a CE/step CE of 25 V.

### 4.6. Image Analyses

#### 4.6.1. SEM Analysis

The FD-BCP sample was mounted on stubs, sputter-coated with a gold-palladium layer, and examined for morphology using a Zeiss GeminiSEM 300 microscope (Carl Zeiss SMT Ltd., Cambridge, UK) at an acceleration voltage of 3 kV.

#### 4.6.2. Fourier Transform Infrared Spectrophotometer (FTIR) Analysis

Using a Perkin Elmer spectrometer (Spectrum 100, Perkin Elmer, Waltham, MA, USA) and Spectrum 100 STD software (Version 3.02.01, Perkin Elmer Inc., Shelton, CT, USA), the FTIR spectrum values of the BCP-E and FD-BCP samples were acquired in the mid-infrared band (4000–650 cm^−1^) with a resolution of 4 cm^−1^ (mean of four scans). Prior to every test, an air background spectrum was captured.

### 4.7. Cytotoxicity Analysis

To investigate the effect of the BCP samples on the cell proliferation of the fibroblast-like cells (L929 cell line), the MTT (3-(4,5-dimethylthiazol-2-yl)-2,5-diphenyltetrazolium bromide) assay was selected. The L929 fibroblast-like cell line was selected as it is commonly used in in vitro cytotoxicity and biocompatibility assessments of biomaterials and bioactive compounds. A 0.5 mg/mL MTT solution was prepared in Dulbecco’s Modified Eagle Medium (DMEM) (EuroClone DMEM Mix F12, Milan, Italy) and sterilized by using a 0.22 μm filter. For the MTT assay, L929 cells were cultured in 96-well plates (Nest Technologies, Sterling, VA, USA) at a density of 1 × 10^4^ cells/well in 100 µL and allowed to adhere overnight in the incubator (Nüve EC160, Ankara, Türkiye; with the incubation conditions of 37 °C, 5% CO_2_, humidified atmosphere). After confirming under a fluorescence microscope (Carl-Zeiss, Axio Observer 3, Oberkochen, Baden-Württemberg, Germany) that cells had reached the appropriate conditions. This step was performed to confirm uniform cell attachment, normal morphology, and appropriate confluency prior to sample treatment. The BCP samples were applied to the L929 cells at different concentrations. The BCP-E and FD-BCP (dissolved in distilled water) samples were filtered for sterilization, and 10, 25, 50, 75, and 100 µg/mL of the BCP-E and FD-BCP samples were applied on the L929 cell line for 24, 48, and 72 h and 10 μL of MTT solution were added to each well to clarify the effects of the BCP samples on cell viability/cytotoxicity at each hour interval. All experimental conditions were performed in quadruplicate as technical replicates. After incubation at 37 °C for 4 h, MTT solution was taken and 75 μL dimethyl sulfoxide (DMSO) was applied to the wells. The wells were gently pipetted until all the dark formazan crystals observed at the bottom were dissolved. A 100 μL of sample was transferred to a 96-well Petri dish and read against 570–670 nm absorbance on an ELISA reader (MultiSkan Go, Thermo Fisher Scientific, Waltham, MA, USA) [52]. Cell viability was expressed as a percentage relative to the untreated control wells. The measurements of untreated control wells and treated wells were analyzed, and IC50 values were calculated via Graphpad Prism—software (Version 8, GraphPad Software Inc., San Diego, CA, USA).

### 4.8. Statistical Analysis and Modeling Procedure

Statistical analyses were performed using SPSS (Version 29.0 IBM SPSS Software, Armonk, NY, USA) and GraphPad Prism software (Version 8, GraphPad Software Inc., San Diego, CA, USA). Each experiment was carried out in triplicate, and the results are expressed as mean ± standard deviation (SD). At a significance level of 5%, statistical differences between groups were assessed by employing Two-way ANOVA with Dunnett’s multiple comparisons test and Independent *t*-test.

Principal Component Analysis and Pearson correlation were performed using OriginPro 2018 (OriginLab Corporation, Northampton, MA, USA).

**Figure 6:**
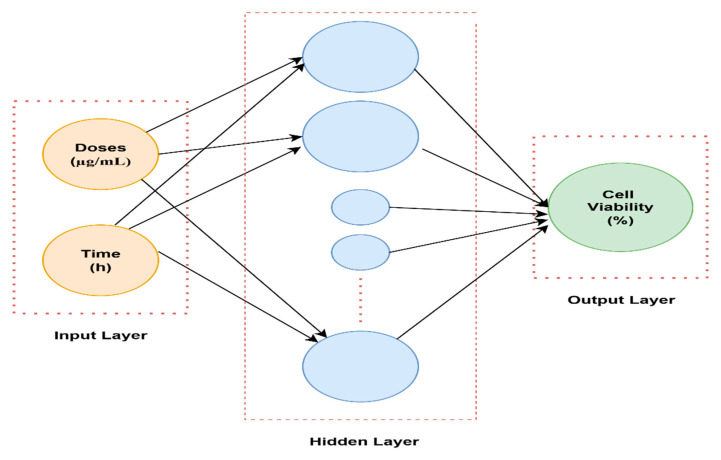
Architecture of the neural network.

The MATLAB graphical user interface (version 2024b) was employed to load the input and output datasets into the Neural Network Toolbox (Figure 6). The input parameters were time (hour) and doses (µg/mL), while the output parameter was cell viability (%). Trainlm is a network training function that automatically optimizes weights and biases to achieve the best nonlinear data fitting using the extremely efficient Levenberg–Marquardt algorithm. In this work, a multilayer feed-forward backpropagation network was used. An adaptation learning rule defined by the Learngdm function was used. Neuronal responses were computed by using the logsig activation function. To avoid overfitting, training was automatically stopped when any one of the termination criteria was met: performance gradient of 10^−7^, six consecutive validation failures, or a maximum of 1000 epochs. Data division for training, validation, and testing was performed in a ratio of 70%, 15%, and 15%, respectively, to ensure reliable prediction performance assessment. One hidden layer was used with five neurons to model nonlinear relationships between inputs and output effectively.

## Figures and Tables

**Table 1:** Antioxidant and color properties of BCP-E and FD-BCP samples.

Parameters/Samples		BCP-E	FD-BCP	Significance *
Antioxidant Properties	TPC (mg GAE/g db)	4.95 ± 0.03	4.46 ± 0.11	0.025
	TMAC (mg/g db)	3.29 ± 0.07	3.25 ± 0.23	0.840
	DPPH (mg TE/g)	7.29 ± 0.05	6.88 ± 0.48	0.350
	ABTS (mg TE/g)	23.90 ± 0.02	23.04 ± 0.48	0.123
Color	L*	3.76 ± 0.20	60.92 ± 0.02	0.000
	a*	9.76 ± 0.39	14.07 ± 0.02	0.000
	b*	2.74 ± 0.49	−4.10 ± 0.01	0.000
	YI	136.43 ± 1.16	7.89 ± 0.03	0.000

*: Independent *t*-test.

**Table 2:** LC-MC analysis results of BCP-E and FD-BCP samples.

Component Number	Component Name in BCP-E Sample	Retention Time (min)	Quantitate Peak (*m*/*z*)	Concentration (ng/mL)
1	Benzoic acid	9.47	121.02950	72.225
2	4-Hydroxybenzoic acid	7.04	137.02442	123.329
3	Salicylic acid	9.45	137.02442	13.891
4	Gallic acid (3,4,5-trihydroxybenzoic acid)	3.45	169.01425	10.351
5	Protocatechuic acid (3,4-Dihydroxybenzoic acid)	5.68	153.01933	856.257
6	3,4-dihydroxybenzaldehyde (Protocatechuic aldehyde)	6.51	137.02442	13.156
7	Vanillin	8.36	151.04007	34.831
8	Coumaric acid (trans-3-Hydroxycinnamic acid)	9.23	163.04007	22.831
9	Caffeic acid	8.19	179.03498	384.855
10	Ferulic acid	9.54	193.05063	166.298
11	Chlorogenic acid	7.92	353.08781	8417.758
12	Quinic acid	0.92	191.05611	1692.212
13	Epigallocatechin gallate	8.19	179.03508	384.741
14	Rhoifolin (Apigenin 7-O-neohesperidoside)	10.94	433.11200	4.486
15	Vitexin (Apigenin 8-C-glucoside)	9.91	431.09837	1.66
16	Luteolin	12.27	285.04046	5.078
17	Diosmetin (Luteolin 4′-methyl ether)	12.96	299.05611	4.137
18	Isoquercitrin (Quercetin 3-glucoside)	10.59	463.08820	251.131
19	Naringenin	11.76	271.06120	10.022
20	Neohesperidin	10.52	461.07269	2.468
21	Liquiritigenin	11.02	255.06628	1567.696
**Component number**	**Component name in FD-BCP sample**	**Retention time** **(min)**	**Quantitate Peak** **(*m*/*z*)**	**Concentration (µg/g)**
1	2,4-dihydroxybenzoic acid (beta-Resorcylic acid)	7.6	153.01933	6.035
2	Chlorogenic acid	7.91	353.08781	89.649
3	Quinic acid	0.88	191.05611	61.773

**Table 3:** Statistical analysis results: Control cell line vs. BCP-E and FD-BCP samples.

Time/Doses		10 μg/mL	*p* Value	25 μg/mL	*p* Value	50 μg/mL	*p* Value	75 μg/mL	*p* Value	100 μg/mL	*p* Value
BCP-E sample	24th h	90.11 ± 20.71	0.4219	84.87 ± 13.73	0.123	80.97 ± 5.17	0.0378	81.83 ± 13.19	0.0499	67.17 ± 9.17	0.0001
	48th h	87.41 ± 13.79	0.2363	68.72 ± 7.81	0.0003	59.93 ± 1.93	<0.0001	59.51 ± 6.01	<0.0001	60.5 ± 4.75	<0.0001
	72nd h	86.92 ± 19.51	0.2099	89.08 ± 22.39	0.3432	76.75 ± 22.74	0.0084	65.9 ± 9.01	<0.0001	63.14 ± 7.33	<0.0001
		**10 μg/mL**	***p*** **value**	**25 μg/mL**	***p*** **value**	**50 μg/mL**	***p*** **value**	**75 μg/mL**	***p*** **value**	**100 μg/mL**	***p*** **value**
FD-BCP sample	24th h	82.99 ± 10.77	0.0932	65.26 ± 5.69	0.0001	77.44 ± 16.67	0.0168	68.51 ± 5.01	0.0006	77.63 ± 10.95	0.018
	48th h	79.74 ± 11.42	0.0359	70.35 ± 19.63	0.0012	69.56 ± 11.02	0.0009	71.07 ± 9.56	0.0016	106.65 ± 3.7	0.7417
	72nd h	92.99 ± 23.47	0.7114	83.51 ± 16.43	0.1075	88.73 ± 23.47	0.3609	94.4 ± 16.92	0.8245	124.24 ± 9.65	0.0094

*p* value at 0.05 by Two-way ANOVA, Dunnet’s post hoc test.

## Data Availability

The original contributions presented in this study are included in the article. Further inquiries can be directed to the corresponding author.
